# Animal-and mineral-based medicines in Gansu-Ningxia-inner Mongolia region, P.R. China: a cross-cultural ethnobiological assessment

**DOI:** 10.3389/fphar.2023.1295806

**Published:** 2023-11-27

**Authors:** Chaoqun Luo, Wenji Zhao, Sha Liu, Mingxia Luo, Tingting Fan, Yongxia Zhao, Yan Ren, Faming Wu, Jian Xie

**Affiliations:** ^1^ School of Pharmacy, Zunyi Medical University, Zunyi, China; ^2^ Sichuan Academy of Grassland Sciences, Chengdu, China; ^3^ Southwest Minzu University, Chengdu, China; ^4^ Guizhou Medical and Health Industry Research Institute, Zunyi Medical University, Zunyi, China

**Keywords:** traditional ethnic medicine, animal and mineral-based medicine utilization, cultural practices in medicine, sustainable development, traditional knowledge

## Abstract

**Introduction:** Traditional animal- and mineral-based medicines are widely used in the Gansu-Ningxia-Inner Mongolia junction zone, a region with diverse ethnic groups and cultures. This study aims to document, conserve, and explore the potential of these medicines for further research and sustainable development of ethnic medicine.

**Methods:** We interviewed 56 informants from different ethnic backgrounds and analyzed their responses quantitatively. Additionally, a comparative analysis with adjacent regions was conducted, providing invaluable contextual insights.

**Results:** The study unveiled a diverse array of traditional medicines in the Gansu-Ningxia-Inner Mongolia junction zone. A total of 47 animal-based medicines were identified, ranging from insects and scorpios to distinctive animal organs. Of notable significance was Moschus, emerging as a pivotal traditional Chinese medicine resource. In parallel, 12 mineral-based medicines were cataloged, procured both locally and from “pharmacies”. Female informants, frequently local herbal practitioners, demonstrated broader knowledge of medicines. The analysis of 13 villages revealed varying perceptions of medicine importance, underscoring the wealth of traditional knowledge. Specific medicines, such as Feng-Mi and Xie-Zi, were widely used and valued in local healthcare practices for their cultural and medicinal benefits.

**Conclusion:** This study provides a comprehensive overview of traditional animal- and mineral-based medicines in the Gansu-Ningxia-Inner Mongolia junction zone. It highlights the need for preserving and applying these practices in a sustainable manner. It also lays a solid foundation for future research on ethnic medicine, which can contribute to the holistic wellbeing of local communities.

## Introduction

Animal and mineral resources have played pivotal roles in traditional medicine for centuries, constituting integral components of natural remedies (Lu, 2013). The roots of this knowledge can be traced back two millennia, when early practitioners of Traditional Chinese Medicine (TCM) meticulously documented the use of animal and mineral-based medicines (Xie, 2015). “Shennong’s Classic of Materia Medica,” the earliest extant Chinese pharmacological treatise, meticulously cataloged 67 animal-based and 41 mineral-based medicines (Wu, 1963). Subsequent works in Chinese pharmacology have further expanded upon these records (Zhang et al., 2022b).

Traditional medicine refers to a medical system developed within a specific cultural and historical context, with its theories and practices based on traditional knowledge, experience, and beliefs (Zhang, 2017). Traditional medicine can be classified into TCM, ethnic medicine, and other forms of traditional medicine. TCM, represented by Chinese traditional medicine, includes herbal medicine, acupuncture, and massage therapy. Ethnic medicine encompasses various traditional medical systems practiced by different ethnic groups, such as Tibetan medicine and Mongolian medicine. The characteristics of traditional medicine include comprehensiveness, holism, individualization, and empiricism (Li, 2017).

In China, traditional medicine has been widely applied and has received significant support and attention from the government. China has established a comprehensive legal framework for TCM, promoting its development and inheritance. Currently, traditional medicine has been widely applied and developed both domestically and internationally. In traditional Chinese medicine, medicines derived from animals, often referred to as “medicines with blood, flesh, and emotions,” (Sun, 1993), are believed to possess greater potency and efficacy than their plant-based counterparts. The Chinese have also developed unique applications for mineral medicines, particularly in utilizing certain toxic minerals, resulting in distinct knowledge systems and safety protocols. For instance, arsenic trioxide (Pi shuang), a highly toxic mineral, is employed for specific medical purposes (Liu et al., 2008). Extensive modern pharmaceutical research has validated both its effectiveness and safety (Mahwish et al., 2010; Su, 2012; Han, 2015; Huang, 2018).

China, home to 56 diverse ethnic groups, each shaped by distinct geographical and environmental factors, boasts a rich tapestry of traditional ethnic medicine deeply ingrained in its culture and history. Over generations, these ethnic groups have accumulated a wealth of medicinal knowledge through the rigors of daily life, production, and warfare, giving rise to unique medicinal cultures. Varied natural environments and resource availability across different regions have led to diverse choices and applications of medicines. For instance, in the Gansu-Ningxia-Inner Mongolia junction area, the local environment and climate conditions have prompted a greater emphasis on the use of mineral and animal-based medicines (Liang, 2015; Garcia-Hernandez et al., 2021). In contrast, other regions, blessed with abundant herbal resources, lean toward herbal medicines (Luo et al., 2019).

Traditional Tibetan medicine, with its extensive history, places significant emphasis on animal and mineral-based remedies (Demar, 2012). Notable traditional remedies, primarily composed of animal ingredients, include “Gangdi Shi Feng Wan,” “Zang Wang Shen Bao Wan,” “Er Shi Wu Wei Lv Xue Wan,” and “Xiong Dan Gao" (Yutuo, 1983). Mineral-based remedies such as “Er Shi Wu Wei Zhen Zhu Wan,” “Er Shi Wu Wei Shan Hu Wan,” “Er Shi Wu Wei Song Shi Wan,” “Qi Shi Wei Shan Hu Wan,” and “Qi Shi Wei Zhen Zhu Wan” are also prominent (Ren and Chen, 2014). The Tibetan Medical Canon meticulously documents up to 258 medicinal substances sourced from animal resources (Dorje, 2007). The preparation of these remedies involves a multistep process, encompassing material selection, cleaning, roasting, grinding, and distillation, culminating in effective medicine (Jia et al., 2015).

Internationally, an increasing number of countries and regions have begun to recognize the value of traditional medicine. Johnson et al. found that kidney diseases may be caused, treated, prevented, improved, or worsened by traditional medicines depending on the setting, the person, and the types, modes, and frequencies of traditional medicine use (Johnson et al., 2022). Grace et al. conducted a structured interview questionnaire with 200 individuals, including patients, hospital visitors and hospital staff, at the main referral hospital in Timor-Leste and found that the use of traditional medicine had become widespread there (Grace et al., 2020). Johnson et al. found through their research that traditional medicine can be a valuable resource for First Nations patients living with diabetes and should be considered a therapeutic modality (Johnson et al., 2022). Additionally, traditional medicine is widely applied in Asian countries. Traditional medicine systems such as Indian Ayurveda (Joshi and Joshi, 2021), Korean medicine (Kim et al., 2016), and Japanese Kampo medicine (Hyun et al., 2019) have deep historical and cultural heritage in various Asian countries. These countries have systematically organized and developed traditional medicine, forming unique medical systems.

The Gansu-Ningxia-Inner Mongolia intersection zone stands as a crucible of Han, Mongolian, and Hui cultures, representing the intersection of ancient nomadic and agrarian traditions in China (Li et al., 2022). This dynamic cultural milieu has engendered a unique system, wherein traditional medicine plays a pivotal role. However, with the march of progress and the influence of both traditional Chinese medicine and modern pharmaceuticals, numerous small-scale medicinal cultures characterized by regional and ethnic distinctiveness face rapid decline (Ma, 2022). This not only entails the loss of medicinal value but also threatens cultural diversity. Hence, it becomes imperative to explore, document, preserve, and pass on this traditional knowledge. Such endeavors hold significant implications for cultural heritage, ethnic identity preservation, the judicious utilization of medicinal resources, and the safeguarding of ecological integrity. Furthermore, they provide invaluable insights for modern pharmaceutical research and the development of traditional medicinal resources. Consequently, this study undertook a systematic examination of animal and mineral-based medicinal resources utilized in the multiethnic regions of the Gansu-Ningxia-Inner Mongolia intersection zone. It sought to systematically compile and organize the traditional knowledge of local residents regarding these medicines, unearthing valuable insights for their rational utilization and development.

## Study area and methods

### Study area

**FIGURE 1:**
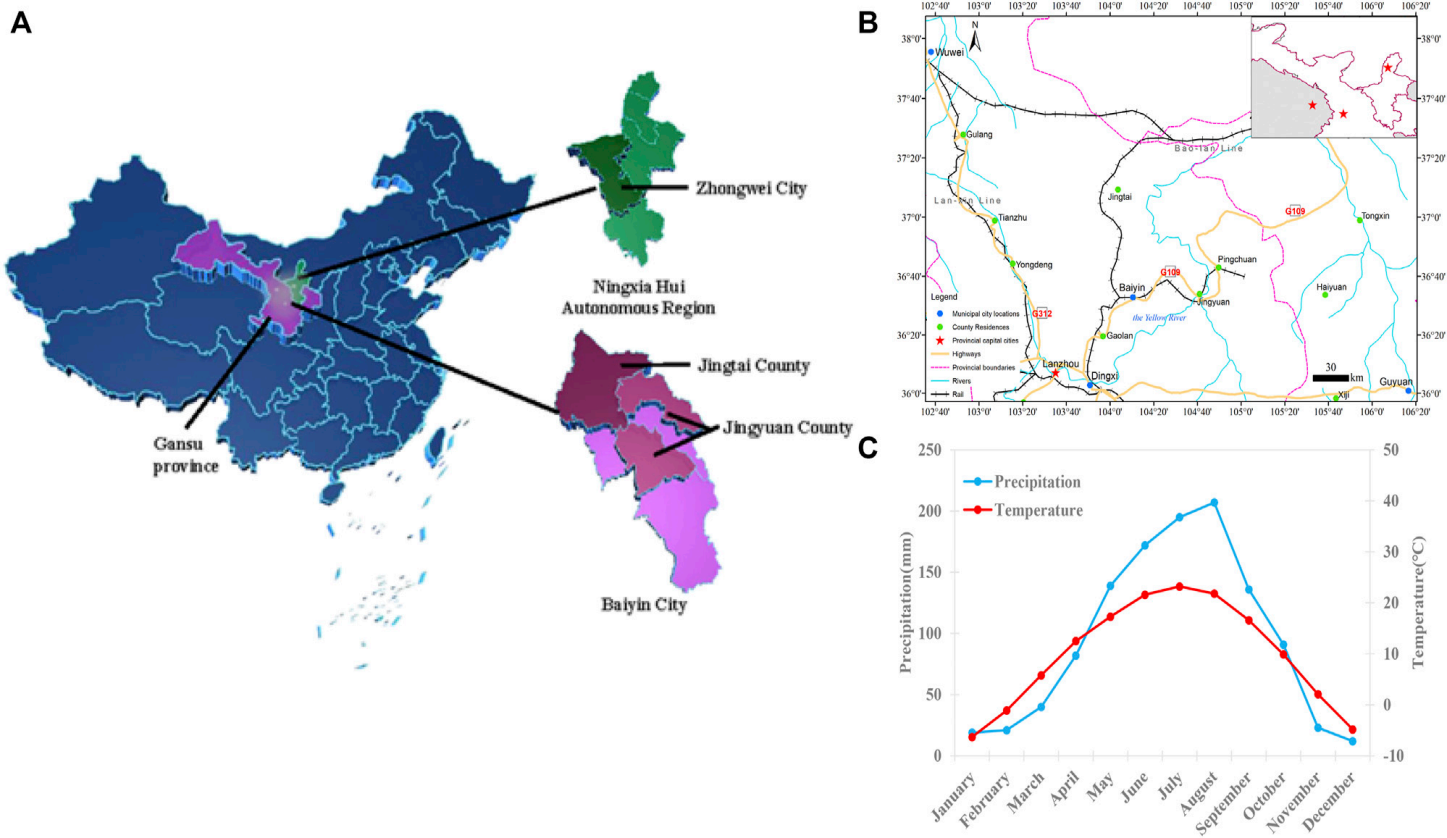
Survey area. **(A)** Jingyuan County, Jingtai County and Zhongwei County belong to an intersection zone of Gansu-Ningxia-Inner Mongolia. **(B)** The traffic situation in the study area. **(C)** The annual average temperature and precipitation.

This study focuses on the Jingyuan-Jingtai-Zhongwei area located at the intersection of Gansu, Ningxia, and Inner Mongolia. The study area lies between 103°33′E to 106°10′E and longitude 36° N to 37°50′N latitude, with elevations ranging from 1,100 to 3,321 m. The average temperature ranges from 7.3°C to 9.5°C throughout the year, with the coldest season typically occurring in winter and the hottest season occurring in summer. The annual precipitation ranges from 180 to 367 mm, with more rainfall occurring in summer and less in winter. The area experiences almost no rainfall during other times. The annual sunshine hours range from 2,696 to 3,796 h, and the region has a temperate continental climate (Figure 1) (Nan et al., 2004). It is located in the upper reaches of the Yellow River, with high terrain in the west and low terrain in the east. It is a gully area of the Loess Plateau, where the gullies intersect with the river beach. This area is a transitional zone between the Loess Plateau and the Tengger Desert and serves as the gateway to the eastern end of the Hexi Corridor (Table 1) (Xu et al., 2007; Gao, 2017).

**TABLE 1:** Basic information of the study areas.

County	Location	Population	Main ethnic groups	Main language	GDP/person	Investigation site	Longitude	Latitude
Jingyuan	E 104°13′-105°15′; N 36°–37°15′	373,100	Han	Chinese	¥26869	Shahe Village Yongxin Township	E 104°35′	N 37°3′
			Hui			Yongxin Township Hassanshan Nature Reserve	E 104°39′	N 37°4′
			Mongolian			Damiao Village	E 104°29′	N 37°12′
			Tibetan			North TanLugou Village	E 104°51′	N 37°12′
						Wulan Town	E 104°41′	N 36°33′
						Dongwan Town Daba Village	E 104°43′	N 36°37′
						Dongsheng Town Xinzhai Village	E 104°58′	N 36°59′
Jingtai	E 103°33′-104°43′; N 36°43′-37°38′	238000	Han	Chinese	¥32142	Caowotan Town Gongjiawan Village	E 104°6′	N 37°17′
			Hui			Luyangshicheng Village	E 104°9′	N 37°8′
			Mongolian			Xiquan Town Santang Village	E 104°3′	N 37°5′
Zhongwei	E 104°17′-106°10′; N 36°06′-37°50′	1075000	Han	Chinese	¥52454	Changshantou Town Pengjian Village	E 105°36′	N 37°21′
			Hui			Dazhanchang Town Dazhanchang Village	E 105°32′	N 37°25′
			Man			Yongkang Town Yongfeng Village	E 105°18′	N 37°29′
			Mongolian					
			Dongxiang					

This region has a rich history dating back to the Western Zhou period in ancient times. During the Han dynasty, it played a crucial role as one of the key passages along the Silk Road and served as an important military stronghold in ancient Northwest China, functioning as a defensive outpost (June 2016). Presently, the area enjoys robust transportation infrastructure, including major highways such as the Lanzhou-Xinjiang and Ningdong Expressways, as well as railway lines such as the Lanzhou-Xinjiang and Lanzhou-Chongqing Railways, facilitating seamless connectivity both within the region and with neighboring provinces and regions (Gao and Dong, 2020; Cao, 2021). The economy of the region leans predominantly on traditional agriculture and animal husbandry, with Han, Hui, Mongolian, Tibetan, Tujia, and other ethnic groups constituting the primary demographic. The population remains relatively modest, owing to the region’s comparatively lower economic development and restricted population mobility, resulting in a pronounced aging population demographic (Bai, 2018). The area’s arid climate translates to relatively meagre vegetation resources, predominantly characterized by desert and grassland flora interspersed with patches of forest, shrubbery, and wetland vegetation. Noteworthy plant species include *R.obinia pseudoacacia* L. (*R. pseudoacacia* L.), *Haloxylon ammodendron* (C. A. Mey.) Bunge ex Fenzl (*H. ammodendron* (C. A. Mey.) Bunge ex Fenzl), *Caragana korshinskii* Kom. (*C. korshinskii* Kom.), *Hippophae rhamnoides* L. (*H. rhamnoides* L.) (Du et al., 2016; Wang et al., 2019), among others. While the area is relatively deficient in animal resources, it is home to diverse wildlife, including species such as *Pseudois nayaur* (Hodgson) (*P. nayaur* (Hodgson)), *Cervus elaphus* Linnaeus (*C. elaphus* L.), *Mellivora capensis* (Schreber) (*M. capensis* (Schreber)) (Liu et al., 2022; Xie, 2022; Liu et al., 2023b). However, it boasts a wealth of mineral resources, encompassing coal, *gypsum fibrosum*, limestone, gold, Yin-Zi, copper, and others (Gao, 2022a; Gao, 2022b; Guan et al., 2022).

### Ethnobotanical information collection

**FIGURE 2:**
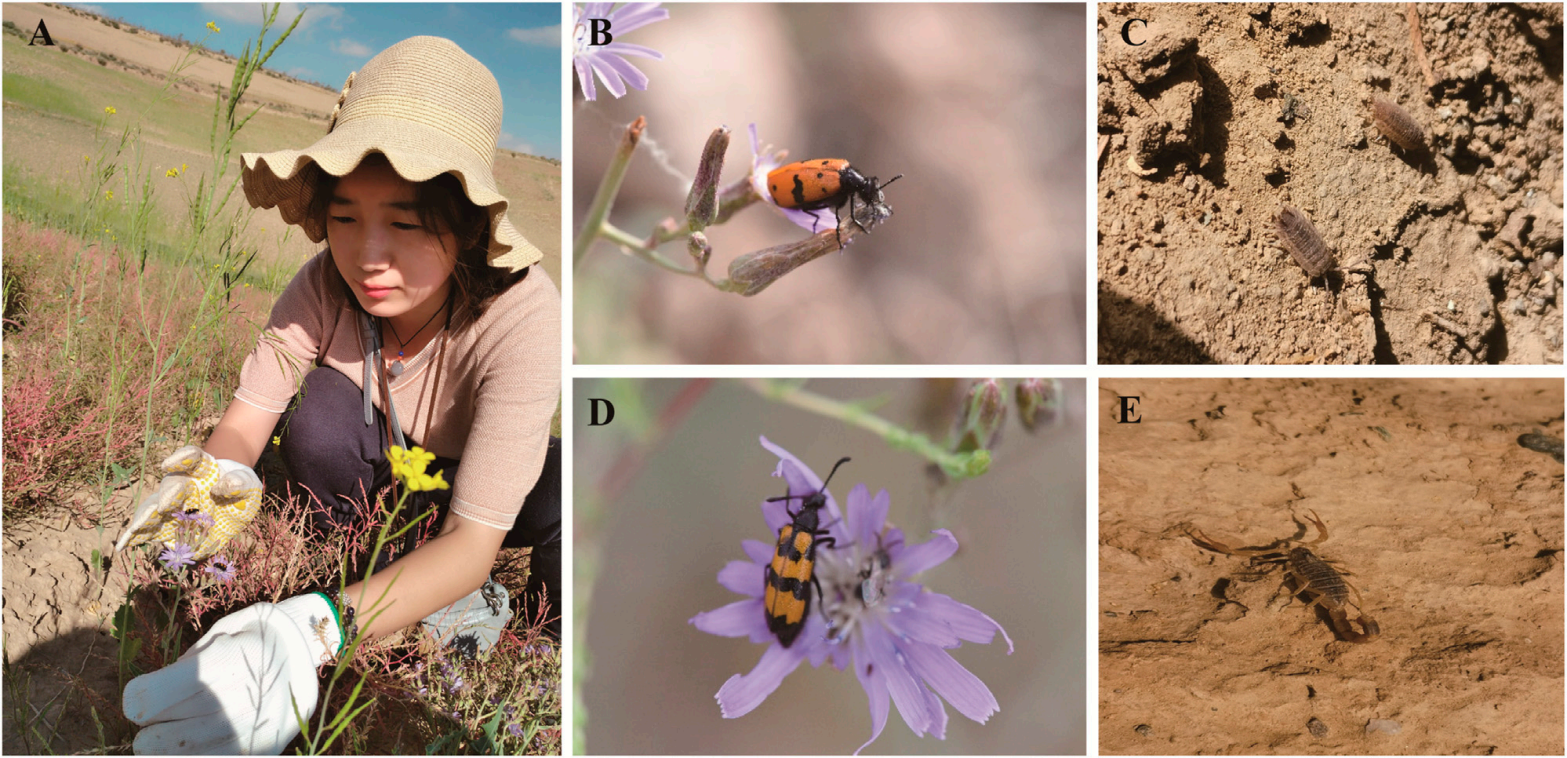
Examples of animal-based medicines. **(A, B, D)** *Mylabris phalerata* is widely used in traditional Chinese medicine with anti-inflammatory, analgesic, and anticancer effects. **(C)** Pillbug is also used in traditional Chinese medicine. It has nourishing, tonifying, and hemostatic effects. **(E)** *Scorpios* is a traditional Mongolian medicine with antispasmodic, anticonvulsant, and antitumor effects.

Ethnobotanical Information Collection During the field survey, we used key informant interviews (Hong et al., 2015), semistructured interviews and participatory rural appraisal (Liu et al., 2014) methods to collect information based on the “5 W+1H” principle (who, what, where when, why, and how) (Pei and Long, 1998). Key informant interviews are a method of interviewing knowledgeable persons as an important part of the research. We selected some traditional healers or herbalists with rich experience and knowledge as key informants and asked them some open-ended questions (Supplementary Table S1) to understand their opinions and suggestions on the use, source, and conservation of animal and mineral medicines. Semistructured interviews are a method of using partly predesigned questions and partly flexible questions to collect qualitative data. In the survey process, we developed an interview guide based on the research objectives and literature review and then asked questions flexibly according to different interviewees and situations to collect information on the use, knowledge, and attitude of animal and mineral medicines. Participatory rural appraisal involves local residents in the research process. We invited local residents to discuss, analyze and evaluate local problems and resources, such as community maps, resource maps, and historical timelines, to express their views and needs on the use, distribution, and threats of animal and mineral medicines and to obtain more local knowledge and experience (Figure 2). During the survey process, we mainly asked the following questions: 1) whether the respondent used any animal or mineral medicine to treat or prevent diseases; 2) if yes, which diseases were the animal or mineral medicine used to treat or prevent; 3) how the animal or mineral medicine was used (including which parts, preparation methods, and use methods); 4) what are the sources of these animal or mineral medicines; 5) how was this knowledge acquired; and 6) what else should be added to this interview. The interviews were conducted in Mandarin (Chinese) or local dialects. During the interview, in addition to written records, audio recordings and photos were taken with the permission of the other party, and the respondents were asked to take us to the adjacent grassland, farmland, or mountain to identify the animal or mineral medicine that they used and provide local colloquial names in Mandarin (Chinese) or local dialects.

Through these survey methodologies, we systematically accumulated and cataloged the traditional knowledge of animal and mineral-based medicines used by local residents and recorded, organized and analyzed the basic information of the informants and the local names, medicinal parts, processing methods, efficacy, and safety of the medicines.

### Data collation and analysis

#### NCSI analysis

We used the National Plant Cultural Significance Index (NCSI) to evaluate the importance of animal and mineral drugs in the surveyed area.

$$\text{NCSI}=\text{FQI}\times \text{AI}\times \text{FUI}\times \text{PUI}\times \text{MFI}\times \text{CEI}\times \text{DSI}\times 10^{-2}$$

In this formula, FQI is the frequency of the quotient index, AI is the availability index, FUI is the frequency of utilization index, PUI is the parts used index, MFI is the multifunctional use index, CEI is the curative effect index, and DSI is the drug safety index (Pieroni, 2000). Each index was established and assigned a score according to the guidelines provided in “Research Methods in Ethnobotany” (Wang and Wang, 2017). The frequency of quotation index (FQI) refers to the number of people among all informants who mentioned a particular plant. The availability index (AI) is divided into four categories: very common (4.0), common (3.0), general (2.0), and uncommon (1.0). The frequency of utilization index (FUI) is divided into six categories: used more than 10 times per year (5.0), used 6-10 times per year (4.0), used 2-5 times per year (3.0), used at least once per year (2.0), used once every 2–3 years (1.0), and not used in the past 5 years (0.5). The Parts Used Index (PUI) is divided into three categories: whole plant (3.0), part of the plant (2.0), and special parts or processed products (1.0). The multifunctional use index (MFI) has a base score of 0 and increases by 1 for each additional use. An item with only one use has a score of 1, and an item with five uses has a score of 5. The Curative Effect Index (CEI) is divided into five categories: excellent (5.0), very good (4.0), good (3.0), fair (2.0), and poor (1.0). The Drug Safety Index (DSI) is divided into five categories: very high (dual-use as food and medicine, score of 5.0), high (safe with no toxic side effects, score of 4.0), moderately high (has some side effects, score of 3.0), moderate (slightly toxic, score of 2.0), and low (highly toxic, score of 1.0).

#### SWOT analysis

SWOT analysis is an analytical framework proposed by Harvard Andrews in 1971 in The Concept of Corporate Strategy. It is a method of systematically identifying strengths, weaknesses, opportunities and threats and proposing strategies to cope with them (Dai, 2000). Among them, strengths are the internal strengths and favorable conditions of the subject of the study, weaknesses are the internal weaknesses and unfavorable conditions of the subject of the study, opportunities are the external opportunities and favorable conditions faced by the subject of the study, and threats are the external threats and unfavorable conditions faced by the subject of the study. An effective strategy should be able to maximize internal strengths and environmental opportunities while minimizing internal weaknesses and environmental threats. Similarly, in traditional medicine, SWOT analysis can help healthcare professionals understand the strengths, weaknesses, opportunities and threats of traditional medicine so that they can formulate appropriate development strategies. Therefore, we use SWOT analysis in this paper (Tang et al., 2018), from the traditional medicine’s own internal and external environmental conditions of the two major aspects of the investigation of some of the traditional medicine of the region’s strengths, weaknesses, opportunities and threats to identify and analyze, and build SWOT strategy quadrilateral matrix, according to the matrix to focus on the factors and can be biased toward the use of the strategy, put forward the corresponding strategic recommendations, from which to find the future development of the strategic direction.

### Specimen identification

**FIGURE 3:**
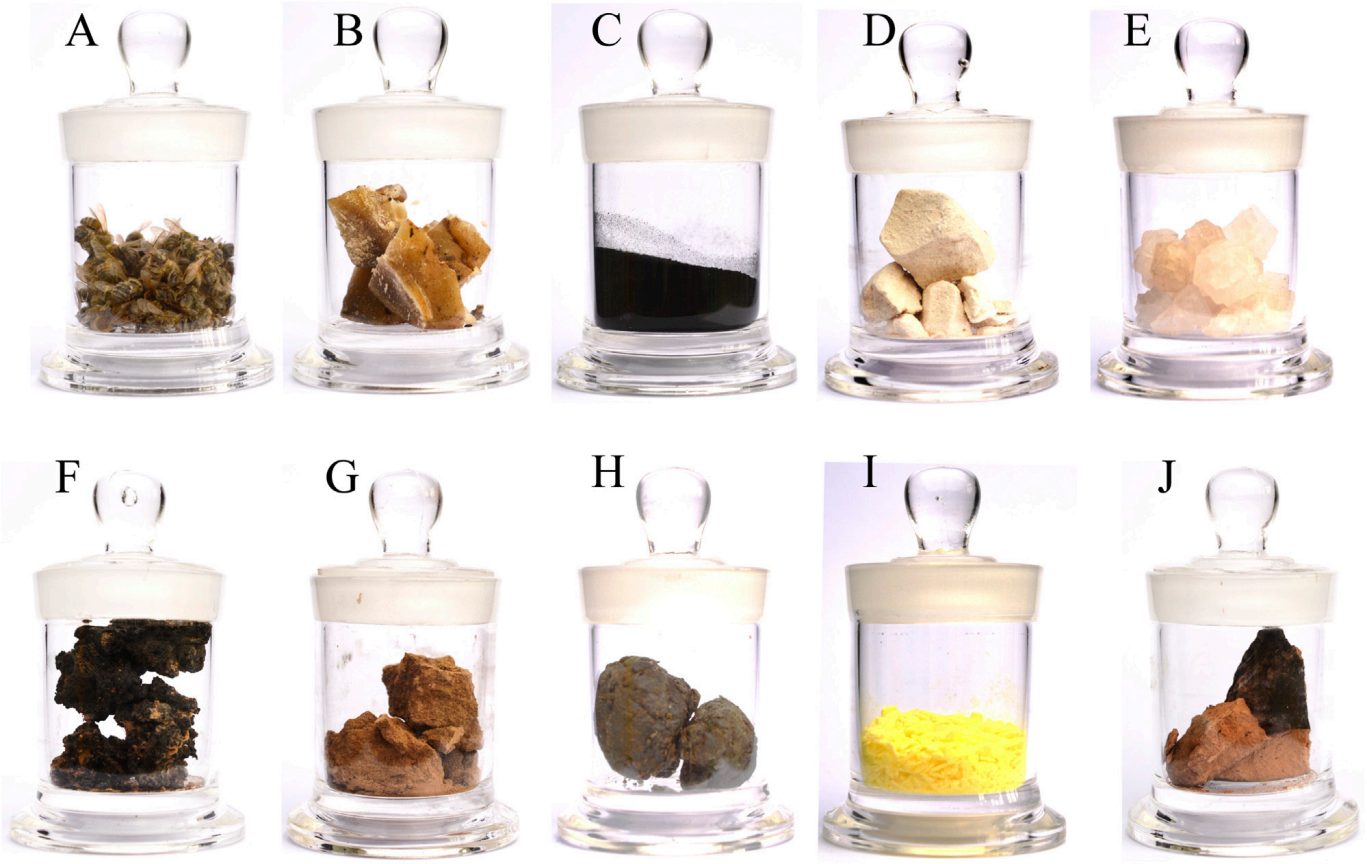
Examples of animal- and mineral-based medicinal specimens collected in the study area. **(A)** *Apis cerana* Fabricius. The dried body of *Apis cerana* Fabricius or *Apis mellifera* Linnaeus. **(B)** *Cera Flava*. Wax secreted by *Apis cerana* Fabricius or *Apis mellifera* Linnaeus. The hive is heated in water and filtered, and the wax is condensed or refined. **(C)** Guo Hui. Ashes were stored on the bottom of the pot after the weed was burned. **(D)** *Gypsum Fibrosum*. For the sulfate mineral gypsum group gypsum, debris and sediment were removed after excavation. **(E)** *Halitum*. Halide salt crystals of the rock salt family. **(F)** Kang-Jing. Tar formed by burning the adobe bed. **(G)** *Loess*. A porous yellow powdery soil with columnar joints formed under dry climatic conditions. **(H)** *Propolis*. A viscous solid gel formed by mixing plant resin collected by the worker bees of the honeybee family, *Apis mellifera* Linnaeus, with secretions from their upper frontal glands and wax glands. **(I)** *Sulfur*. Natural sulfur of the sulfur group of natural elemental minerals is mined, heated and melted to remove impurities. **(J)** *Terra Flava Usta*. A scorched yellow clod in the center of the bottom of an earthen stove for burning wood or weeds.

The sources of the animal and mineral-based medicines were collected during the investigation by referencing “The Illustrated Book of Chinese Medicinal Animals” (Li et al., 2014), “Mineral Medicine Authenticity Illustration and Application” (Gao, 2014), and “Important Medicinal Insects of China” (Yang, 2015). Voucher specimens (bottle specimens) were meticulously prepared and preserved. Collected data were meticulously sorted and analyzed in alignment with the research objectives, with graphical representations generated. The voucher specimens were archived in the Chinese Medicinal Specimen Museum at the School of Pharmacy, Zunyi Medical University (Figure 3).

This study received ethical approval from the Animal Ethics Review Committee of Zunyi Medical University (ZMU21-2,203-291) and the Medical Ethics Committee of Zunyi Medical University ([2022]1-291).

## Results

### Characteristics of informants

**FIGURE 4:**
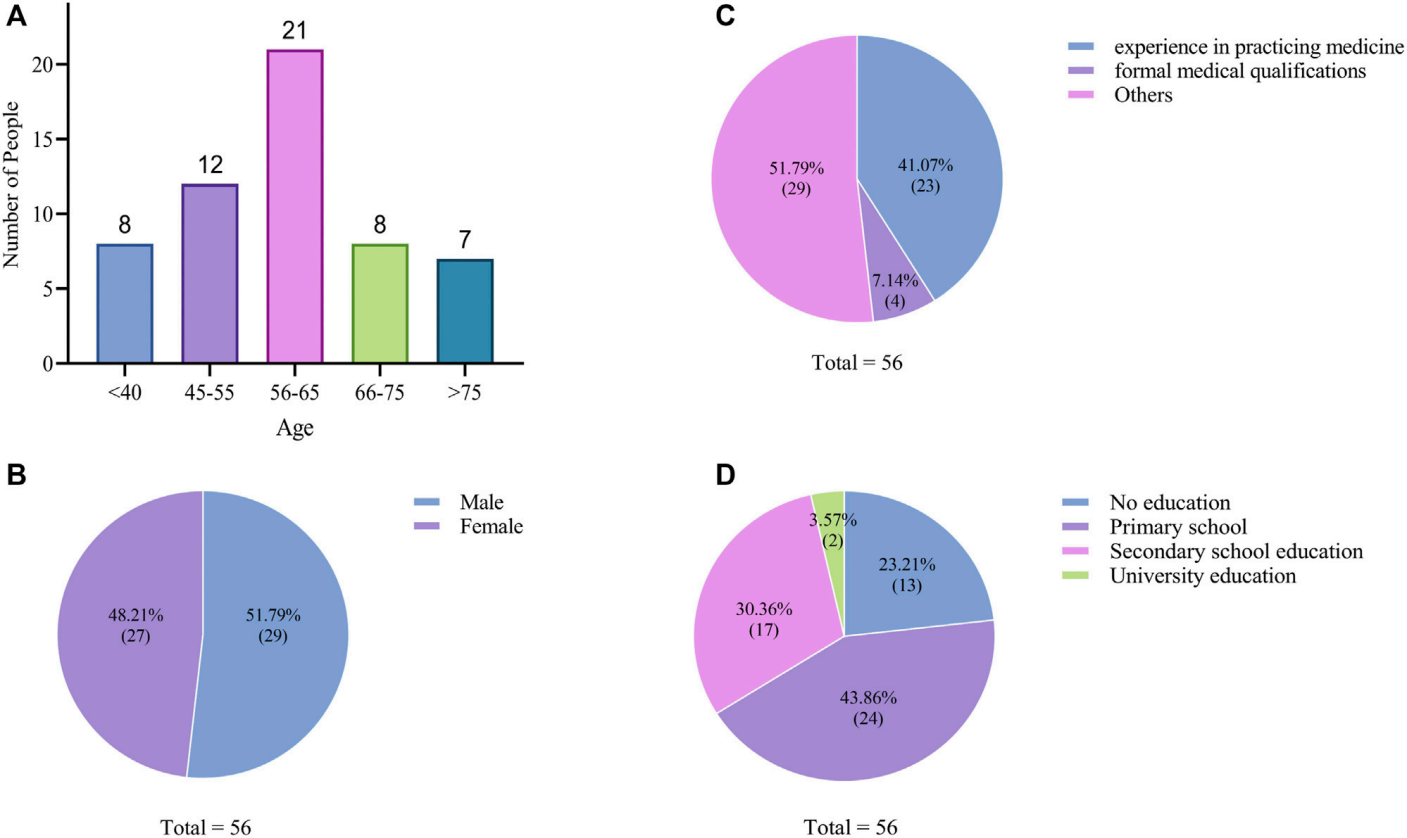
Demographic profile of informants. **(A)** Age structure of informants. **(B)** Gender ratio of informants. **(C)** Level of experience of ethnic medicine among informants. **(D)** Education level of informants.

This study incorporated data from fifty-six informants who provided substantive information. Descriptive analysis was conducted to delineate their age, gender, and ethnic composition. The age range of informants spanned from 35 to 84 years, with eight participants below 40, twelve between 45 and 55, twenty-one between 56 and 65, eight between 66 and 75, and seven above 76 years of age (Figure 4A). The gender distribution was nearly equal, with twenty-nine male and twenty-seven female informants (Figure 4B). Among these participants, twenty-three had prior experience in medical practice, with four holding formal medical qualifications (Figure 4C). All informants had experience using or being treated with animal or mineral medicines (individuals unable to provide valid information were excluded). They were thoroughly interviewed, and their responses were meticulously recorded. Prior to the interviews, informed consent was obtained from all informants, who also affixed their signatures to the consent forms.

According to the survey, women demonstrated an average familiarity with 28.23 types of animal or mineral-based medicines, while men were acquainted with approximately 14.51 kinds. Consequently, in comparison to men, women exhibited broader knowledge of animal or mineral medicines. This phenomenon deviates from findings in other studies, and we posit that it may be attributed to the prevalence of female local traditional herbal doctors (shamans).

Different age groups had different knowledge of animal or mineral medicines. The demographic most inclined to employ these medicines for treating ailments comprised individuals above 45 years old (85.71%). Research has indicated that older individuals tend to possess deeper comprehension and acceptance of traditional medicines (Bouasla and Bouasla, 2017; Lee et al., 2019; Huang et al., 2022). This can be ascribed to the underdevelopment of modern medicine decades ago, where local traditions predominantly relied on ethnic medicine. Furthermore, this cohort might be less inclined to trust in ethnic medicine due to inheritance issues, deeming it superstitious.

With regard to education levels, of the fifty-six informants, thirteen had received no formal education (23.21%), twenty-four had attained primary school education (43.86%), seventeen possessed secondary school education (30.36%), and two had acquired university education (3.57%). The majority exhibited lower educational attainment (Figure 4D). This pattern suggests that knowledge regarding ethnic medicine application primarily circulates among individuals with limited formal education. It is surmised that informants with lower educational backgrounds harbor greater interest in traditional medicine and engage more frequently with the natural environment. Conversely, those with higher education levels evince reduced interest in traditional medicine, owing to prolonged exposure to modern educational systems, resulting in fewer encounters with medicinal plants and local medicine-related knowledge. Analogous results reported in other studies highlight that individuals with lower educational attainment, including illiterate individuals, possess greater proficiency in using medicinal plants compared to intellectuals (Ahmad et al., 2017; Miara et al., 2018).

### Animal and mineral-based medicine resources

A total of 47 types of animal medicine and 12 types of mineral medicine used by local residents were identified in the surveyed area (Table 2). Among the 20 types of animal medicine derived from wild animals, the majority were sourced from small insects such as Xie-Diban, Tui-Louzi, Shi-Panniu, and *Scorpio*. The most precious among the wild animal medicines was *Moschus*, which was also a valuable traditional Chinese medicine material, but its original wild animal had basically become extinct in the area. The *Moschus* we investigated had been treasured by local residents for at least 50 years. Currently, the *Moschus* used in Chinese medicine and other ethnic groups is mostly synthetic or obtained through artificial breeding. In addition, Wu-Yazui, Lang-Mao, Tu-Mao, and He-Magezao were rarely used by other ethnic groups.

**TABLE 2:** Basic information of animal-mineral medicines.

Local name	English name	Source	Use part	Processing method	Ethnopharmacological employment/use	Method of application	Voucher numbers^4^
Zhu-Kudan	Pig’s bitter gall	*Sus scrofa* domestica Brisson (*S. scrofa* domestica Brisson)	Guts	Direct use of fresh products	Redness of the eyes/Weeping	Washing/Drops in the eyes/Small amount for internal use	2023-DK-012
Feng-Mi	Honey	*Apis cerana* Fabricius (*A. cerana* Fabricius)/*Apis mellifera* Linnaeus (*A. mellifera* L.)	Products	Direct acquisition	Mouth ulcers/Constipation	Topical application/Internal use	2023-DK-001
Feng-La	Beeswax	*A. cerana* Fabricius/*A. mellifera* L	Products	Direct acquisition	Various inflammatory diseases	Topical application/Decoction with water	2023-DK-021
Feng-Fang	Nidus vespae	*A. cerana* Fabricius/*A. mellifera* L	Products	Direct acquisition	Itchy skin	Decoction with water	2023-DK-022
Feng-Jiao	Propolis	*A. cerana* Fabricius/*A. mellifera* L	Products	Direct acquisition	Skin diseases	Topical application/Decoction with water	2023-DK-029
Mi-Feng	Bee	*A. cerana* Fabricius/*A. mellifera* L	All	Direct use of fresh products	Rheumatoid arthritis	Lure bees to sting the affected area	2023-DK-011
Hua-Banmao	Mylabris	*Mylabris phalerata* Pallas (*M. phalerata* Pallas)/*Mylabris cichorii* Linnaeus (*M. cichorii* L.)	All	Drying after scalding	Mad dog bite	Powdered and applied externally	2023-DK-033
Xi-Querou	Magpie’s Meat	*Pica pica* (*P. pica*)	Flesh	Kill and clean feathers and guts after capture	Deficiency syndrome^1^/Diabetes	Stew	——
Wu-Yazui	Crow’s beak	*Corvus sp*. (*Corvus sp*.)	Beak	Kill and clean feathers and guts after capture	Cough with deficiency-heat^2^	Stew	——
Tu-Mao	Rabbit hair	*Lepus capensis* Linnaeus (*L. capensis* L.)	Fur	Cutting	Mental illness	Make lotus wear with other medicines	2023-DK-031
Xie-Diban	Pillbug	*Porcellio* sp. (*Porcellio sp*.)	All	Collected and scalded, dried	Traumatic fractures	Grind and swallow	2023-DK-014
She-Dan	Snake gall	*Serpentiformes*	Guts	Fresh use/Shade dried	Redness of the eyes/Weeping	Washing/Drops in the eyes/Small amount for internal use	2023-DK-028
She-Tui	Snake skin cast off during molting	*Serpentiformes*	Skin	Removal of impurities after field collection	Mumps	Fried with eggs/Decoction with water	2023-DK-019
She	Snake	*Serpentiformes*	All	Wild caught and killed to make wine	Various kinds of rheumatoid arthritis	Infusion of wine for internal or external use	2023-DK-010
Tai-Yanggao	Fetal lamb	*Capra hircus* Linnaeus (*C. hircus* L.)/*Ovis aries* Linnaeus (*O. aries* L.)	All	Collection of aborted stillbirths	Deficiency syndrome	Medicinal food/Direct consumption	——
Yang-Nai	Goat’s colostrum	*C. hircus* L./*O. aries* L	Breast milk	Direct use of fresh products	Redness of the eyes/Weeping	Washing	——
Yang-Bian	Penis and testis of a goat	*C. hircus* L./*O. aries* L	Penis	Collection at the time of slaughter	Enhance male sexual function/Male infertility	Soup making/Infusion of wine	——
Yang-Shen	Goat’s renal	*C. hircus* L./*O. aries* L	Testicles	Collection at the time of slaughter	Enhance male sexual function/Male infertility	Soup making/Infusion of wine	——
Yang-Jiao	Goat’s horn	*C. hircus* L./*O. aries* L	Horns	Collection and crushing at the time of slaughter	Eye diseases/Fverish	Decoction with water	2023-DK-015
Tai-Pan	Placenta	*Homo sapiens* Linnaeus (*H. sapiens* L.)	Placenta	The placenta of a woman in delivery, baked in tiles	Female body weakness	Grind and swallow	2023-DK-005
Zuo-Tu	Loess	*H. sapiens* L	Blood	The blood-sucking soil formed by absorbing the bleeding of women during childbirth with loess	Weakness or blood deficiency due to various diseases	Soak in warm water, strain and drink	——
Ren-Nai	Breast milk	*H. sapiens* L	Breast Milk	Direct use of fresh products	Redness of the eyes/Weeping	Washing/Drops in the eyes	——
Tou-Fa	Hair	*H. sapiens* L	Hair	Cutting	Baby cries	Use female hair for male children and male hair for female children, decoction in water/Making a purse to wear	2023-DK-025
Tong-Ziniao	Children’s urine	*H. sapiens* L	Urine	Direct acquisition	Various kinds of deficiency fever	Medicine primer	——
Ban-Mao	Cantharides	*Lytta caraganae* Pallas *(L. caraganae* Pallas)	All	Drying after scalding	Dog bites	Powdered and applied externally	2023-DK-030
Shi-Panniu	Dung beetle	*Geotrupidae*	All	Drying after scalding/Baking on tiles	Cancer	Grind and swallow	2023-DK-016
Cao-Chong	Paramecium caudatum	*Holotrichia diomphalia* Bates (*H. diomphalia* Bates)	All	Add salt and then dissolve the water	Burns and scalding/Ulcers	Topical application	2023-DK-024
Tuan-Zhu	Honey badger	*M. capensis* (Schreber)	Fat	Collecting fat for storage in clay pots after trapping	Frostbite/Water and fire burns	Topical application	——
Ma-Que	Sparrows	*Passer montanus* (Linnaeus) (*P. montanus* L.))	Flesh	Killed after trapping	Postpartum or post-illness weakness^3^	Cooked or baked and eaten	——
Lü-Fen	Feces of donkey	*Equus asinus* Linnaeus (*E. asinus* L.)	Feces	Direct use of fresh products	Dog bites	Topical application	2023-DK-035
Lü-Rou	Flesh of donkey	*E. asinus* L	Flesh	Direct use of fresh products	Deficiency syndrome	Medicinal food/Direct consumption	——
She-Xiang	Moschus	*Moschus berezovskii* Flerov (*M. berezovskii* Flerov)/*Moschus sifanicus* Przewalski (*M. sifanicus* Przewalski)	Products	Put them into a porcelain vase and put a few sewing needles to seal and raise the needles	Swelling and poisoning	Draw circles with a needle around and on the surface of the lump	2023-DK-036
Lang-Mao	Wolf hair	*Canis lupus* Linnaeus (*C. lupus* L.)	Fur	Collecting in the field	Foreign objects in the eyes	Use Lang-Mao to hook foreign objects out	——
He-Magezao	Tadpoles	*Rana nigromaculata* Hallowell (*R. nigromaculata* Hallowell)	All	Drying after scalding	Cancer	Decoction with water	——
Tui-Louzi	Ant lion larva	*Myrmeleon micans* Mac Lachlan *(M. micans* Mac Lachlan)	All	Sifted through a sieve and scalded to death in hot sand to dry	Cancer	Grind and swallow	2023-DK-013
Ji-Neijin	Membranes of chicken gizzards	*Gallus gallus* domesticus Brisson (*G. gallus* domesticus Brisson)	Stomach	Collected and shade dried	Indigestion	Take directly after mashing/Serve with rice and vegetables	2023-DK-004
Ji-Dan	Eggs	*G. gallus* domesticus Brisson	Egg	Direct use of fresh products	Water and fire burns	External application of egg white	2023-DK-009
Niu-Bian	Bull’s penis	*Bos taurus* domesticus Gmeli (*B. taurus* domesticus Gmeli)	Penis	Collection at the time of slaughter	Enhance male sexual function/Male infertility	Soup making/Infusion of wine	——
Niu-Shen	Ox kidney	*B. taurus* domesticus Gmeli	Testicles	Collection at the time of slaughter	Enhance male sexual function/Male infertility	Soup making/Infusion of wine	——
Niu-Jiao	Ox horn	*B. taurus* domesticus Gmeli	Horns	Collection and crushing at the time of slaughter	Eye diseases/Fverish	Decoction with water	2023-DK-026
Gou-Bian	Dog’s penis	*Canis lupus* familiaris Linnaeus (*C. lupus* familiaris L.)	Penis	Collection at the time of slaughter	Enhance male sexual function/Male infertility	Soup making/Infusion of wine	——
Gou-Shen	Dog kidney	*C. lupus* familiaris L	Testicles	Collection at the time of slaughter	Enhance male sexual function/Male infertility	Soup making/Infusion of wine	——
Gou-Xue	Dog’s blood	*C. lupus* familiaris L	Blood	Direct use of fresh products	Mental illness	Internal use	——
Ge-Zi	Pigeons	*Aplopelia* Bonaparte (*A.* Bonaparte)	Flesh	Killed after trapping	Postpartum or post-illness weakness	Soup making	——
Ma-Yi	Ant	*Polyrhachis vicina* Roger (*P. vicina* Roger)	All	Drying after scalding	Male sexual dysfunctions	Infusion of wine for internal use	2023-DK-018
Xie-Zi	Scorpions	*Buthus martensii* (Karsch) (*B. martensii* (Karsch))	All	Drying after capture by boiling water	Rheumatic diseases	Decoction with water/Infusion of wine for internal or external use	2023-DK-002
Lai-Guazi	Toad	*Bufo bufo* gargarizans Cantor (*B. bufo* gargarizans Cantor)/*Bufo melanostictus* Schneider (*B. melanostictus* Schneider)	All	Kill after capture and shade dry	Cancer	Decoction with water	——
Zhu-Sha	Cinnabaris	HgS	——	Grinding powder	Mental illness	Decoction with water	2023-DK-020
Xin-Hong	Arsenolite	As_2_O_3_	——	Grinding powder	Skin diseases	External use	——
Shi-Gao	Gypsum Fibrosum	CaSO_4_·2H_2_O	——	Grinding powder/Cauterize thoroughly and then grind	Fverish/Skin diseases/Water and fire burns	Decoction in water for the treatment of fever/Apply externally when treating skin diseases and water and fire burns	2023-DK-017
Li-Toutu	Plow soil	SiO_2_/Al_2_O_3_	——	Grinding powder	Mental illness	Decoction with water	2023-DK-023
Tie-Xiu	Rust	Fe_2_O_3_	——	Grinding powder	Mental illness	Decoction with water	2023-DK-027
Zao-Xintu	Oven earth	H_2_SiO_3_/Al_2_O_3_/Fe_2_O_3_	——	Grinding powder	Gastrointestinal dseases	Decoction with water	2023-DK-008
Guo-Hui	Pot bottom ash	H_2_SiO_3_/Fe_2_O_3_/CaO/MgO	——	Grinding powder	Gastrointestinal dseases/Hemorrhage	Decoction with water/Topical application	2023-DK-006
Kang-Jing	Tar	——	——	Grinding powder	Parasites/Skin diseases	Decoction with water/Topical application	2023-DK-032
Huang-Tu	Loess	H_2_SiO_3_/Al_2_O_3_/Fe_2_O_3_	——	Grinding powder	Bleeding from trauma	Topical application	2023-DK-003
Da-Qingyan	Carnallite	NaCl	——	Grinding powder	Skin diseases/Mouth ulcers	External use/Dissolve into water for washing	2023-DK-007
Liu-Huang	Sulphur	S	——	Grinding powder	Skin diseases	External use	2023-DK-034
Yin-Zi	Silver	Ag	——	Forming tools	Scalp skin diseases	Comb your hair regularly with a silver comb	——

Notes: ^**1**^Deficiency syndrome refers to the condition of weakness in the human body, usually caused by insufficient Qi and blood, weak Yang Qi, and other reasons. The manifestations of deficiency syndrome include fatigue, lack of strength, mental fatigue, poor appetite, and pale complexion; ^**2**^Cough with deficiency-heat refers to the symptoms of cough accompanied by deficiency-heat. Deficiency-heat refers to excessive Yang Qi and insufficient Yin fluids in the body, leading to heat syndrome. The manifestations of cough with heat deficiency include dry cough, dry and painful throat, and dry mouth and tongue; ^**3**^Postpartum or postillness weakness refers to the weakened state of the body in women after childbirth or long-term illness. This kind of weakness is usually caused by excessive postpartum bleeding, excessive physical exertion, or depletion of physical strength due to prolonged illness. The manifestations of postpartum or postillness weakness include fatigue, lack of energy, poor appetite, and weight loss. ^**4**^Specimens such as Lang-Mao, Zuo-Tu and Tuan-Zhu are usually difficult to obtain, and specimens such as Ren-Nai, Gou-Xue and Yang-Nai cannot be preserved for a long time, so no specimens have been collected, and are uniformly represented by "-".

Of the twenty-two animal-based medicines sourced from domestic animals, the majority comprised organs and physiological or pathological products, categorized into organ, bone, and whip subtypes. Notably, Lü-Fen and Gou-Xue stood out among domestic animal-based medicines. Local residents applied heated Lü-Fen externally to treat dog bites, while Gou-Xue was employed to address mental illness, accompanied by an element of superstition (local residents believed Gou-Xue possessed exorcistic properties). Furthermore, human-derived medicines were extensively employed locally. Zuo-Tu was not reported for use in other regions or ethnic groups. While traditional Chinese medicine also incorporates Tou-Fa, it is processed into *Crinis carbonisatus* for application, diverging significantly from local utilization.

**FIGURE 5:**
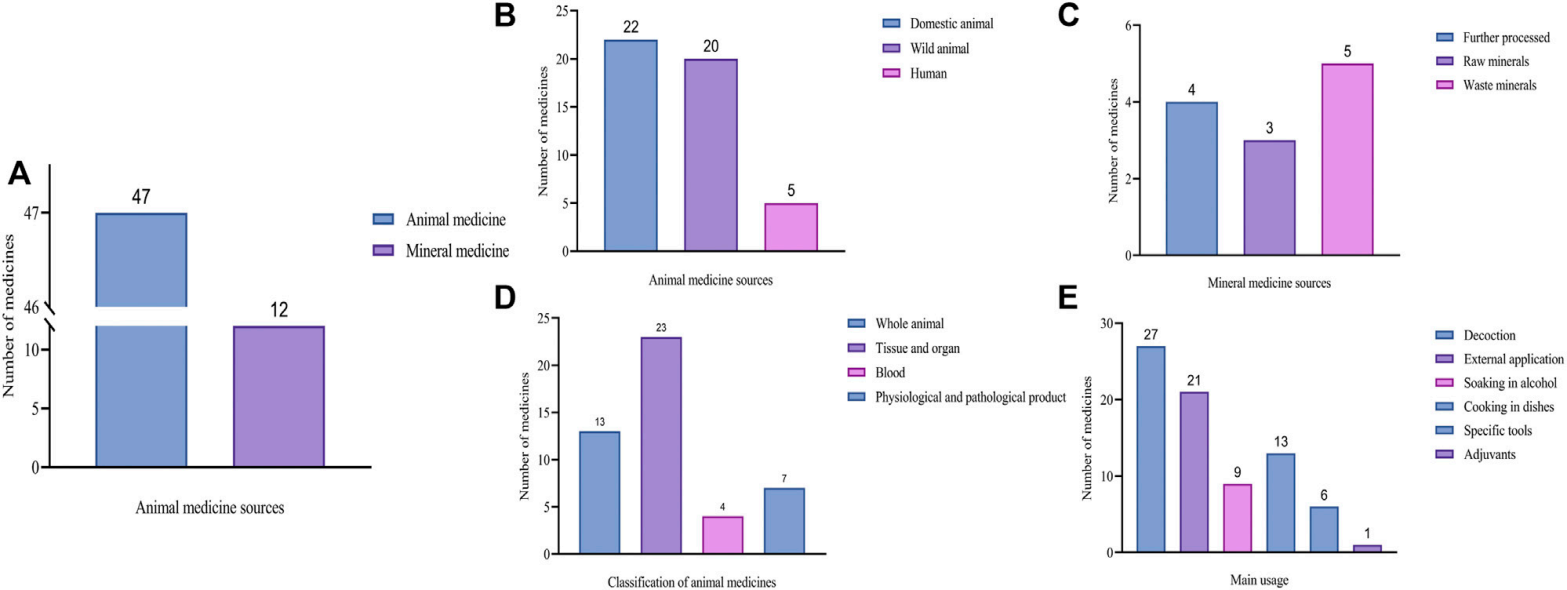
Source, classification and use of animal- and mineral-based medicines documented in the Gansu-Ningxia-Inner Mongolia intersection zone. **(A)** Sources of medicines. **(B)** Animal medicine sources. **(C)** Mineral medicine sources. **(D)** Classification of animal medicines. **(E)** The main usage of animal and mineral medicines.

A smaller number of mineral-based medicines (12 types) were identified, with *Cinnabaris* and *Realgar* being the most commonly used, despite not being locally produced. These minerals were primarily used for treating mental and skin disorders (Figure 5). *Cinnabaris* and *Realgar* (also called Xin-Hong by local residents) were widely used in various exorcism charms.

### Production and use of animal and mineral-based medicine

Local residents primarily rely on gathering animal medicines from insects and other small creatures found in the wild. These creatures are typically captured and processed in abundance, such as the Tui-Louzi, *Mylabris*, *L. caraganae* Pallas, and *Scorpio*. Additionally, organ, bone, and horn medicines are obtained during animal slaughter or from raised animals, including the widely used digestive aid and stomach tonic, *Galli Gigerii* Endothelium Corneum (*G. Gigerii* Endothelium Corneum) extract. In terms of animal organ use, local residents’ understanding is influenced by the traditional Chinese medicine concept of “supplementing form with form,” particularly regarding the extensive use of animal sexual organs to address infertility.

These medicines are usually collected, ground into a powder, and then dried for preservation. Among these, *scorpios* are the only animal medicine purchased as a commercial product by locals. However, they are now under strict protection by the Chinese government, and capturing them is strictly prohibited. Despite this, this region boasts a history of being a renowned traditional source of *Scorpios*. Capturing and selling wild *Scorpios* used to be a vital source of income for local residents. Currently, however, wild Scorpio resources are extremely scarce, and attempts at breeding *Scorpios* have yielded unsatisfactory results.

Mineral-based medicines fall into two categories: imported and locally produced. Minerals such as *Cinnabaris*, *Sulfur*, Yin-Zi, and *Gypsum Fibrosum* are predominantly procured from “pharmacies” (these “pharmacies” are not the conventional ones that sell regulated and standardized medicines. Rather, they are clinics run by barefoot doctors in rural China, who are traditional healers who practice ethnic medicine. These “pharmacies” mainly sell the medicines that barefoot doctors collect or obtain from nature and prepare or process according to their own inheritance or experience.) and are usually kept in small reserves within households. Locally produced minerals such as Guo-Hui, Kang-Jing, *Loess*, and *Terra Flava* Usta are commonly integrated into daily life and are typically used immediately after collection. The history of using these medicines is challenging to trace back, as local residents consider it an instinctive habit. For instance, using *loess* to stop bleeding is perceived as a natural response. When injured and bleeding, *loess* is the most readily available and effective remedy found in the wild, and experience has shown that it possesses a significant hemostatic effect. Consequently, this practice is unanimously followed. Kang-Jing, distinct from this ethnic group, is employed for treating skin and parasitic diseases (including toothaches, which local residents attribute to parasites within the teeth). The underlying principle of its efficacy remains unclear. Furthermore, local residents primarily use mineral medicines individually and rarely combine several medicines.

Local residents use animal-based medicines to address diseases characterized by deficiency, cancer, and eye ailments. Nine primary medicines are employed to address deficiencies, embodying the characteristic of animal medicines as “products of flesh and blood with emotions.” Locals firmly believe that most chronic conditions can be ameliorated or cured by treating deficiencies with tonics. For instance, they hold that Tai-Yanggao (the deceased fetus of a goat or sheep from childbirth) and human placenta uterina possess potent tonic effects and are frequently used for postpartum and postillness recuperation.

Another category of medicines, referred to as “whip medicine” (involving animal penis or testicles), is used to address male sexual dysfunction and infertility. This category encompasses seven distinct types and reflects the principle of “consuming what you lack.” These animal parts, combined with specific plant medicines like *Epimedii Folium* and *Cynomorii Herba*, which are widely employed in treating male sexual dysfunction and infertility, are often stewed and ingested. Some middle-aged and elderly men opt to soak these medicines in wine to enhance or sustain their sexual prowess. The use of penis and testicles aligns with modern pharmacology’s bioactivity, while the utilization of kidneys aligns with traditional Chinese medicine’s theory that kidneys store essence and govern reproduction.

**FIGURE 6:**
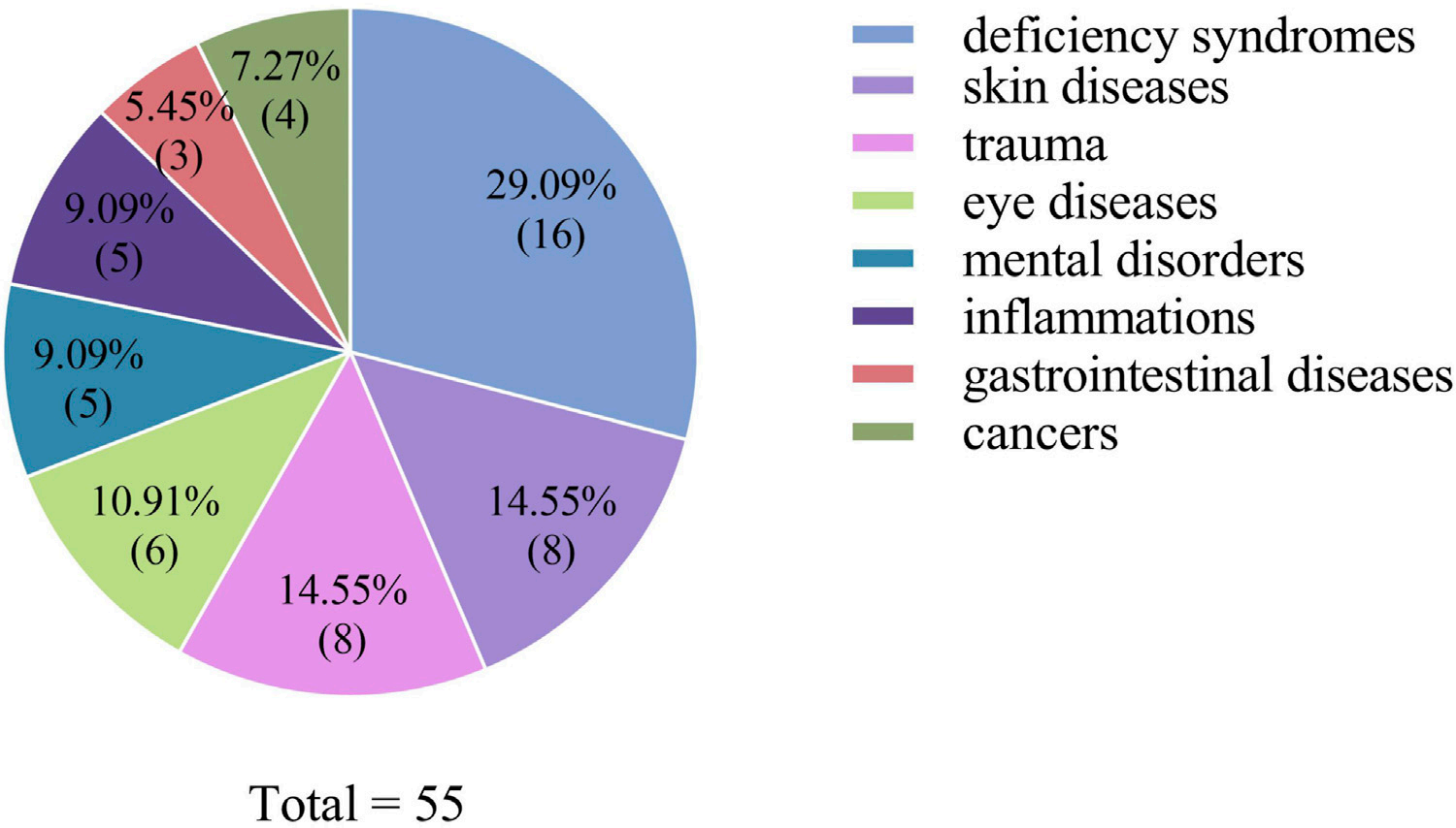
Diseases mainly treated by mineral medicines in the Gansu-Ningxia-Inner Mongolia intersection zone.

Mineral-based medicines are mainly used to treat two types of diseases: mental illness and skin diseases or injuries. For example, Li-Toutu or Tie-Xiu may have a magnetic effect on mental illness (a traditional local understanding and use of certain mineral medicines, which are believed to cure mental illness by magnetically attracting and removing pathogenic factors from the body), and *Loess*, *Sulfur*, and Yin-Zi are mainly used for their astringent, hemostatic, and bactericidal properties to treat skin diseases or injuries (Figure 6).

### Evaluation of the importance of animal and mineral-based medicines used by local residents

An assessment of the significance of 47 traditional animal medicines and 12 mineral medicines was carried out utilizing the National Plant Cultural Significance Index (NCSI) as the evaluative metric. These 59 traditional medicines were categorized. The first tier of paramount importance (NCSI >100) encompasses 13 medicines, comprising 11 animal-based medicines and 2 mineral-based medicines. Notable representatives in this category encompass Feng-Mi, *Scorpio*, *G. Gigerii* Endothelium Corneum, *Loess*, *Placenta uterina*, and *Halitum*. These medicines are the most widely recognized and easily accessible among local residents.

The second important echelon (100 > NCSI ≥10) includes 21 medicines, with 19 being animal-based medicines and 2 being mineral-based medicines. Prominent medicines in this grouping include Zhu-Kudan, Tui-Louzi, Xie-Diban, and Shi-Panniu. These medicines are primarily sourced from commonplace insects and small creatures in the local environment, as well as domesticated animals that are comparatively easy to obtain.

The third significant tier (10 > NCSI ≥1) encompasses 19 animal and mineral medicines, of which 7 are mineral medicines. This category exhibits the highest prevalence of mineral medicines.

**FIGURE 7:**
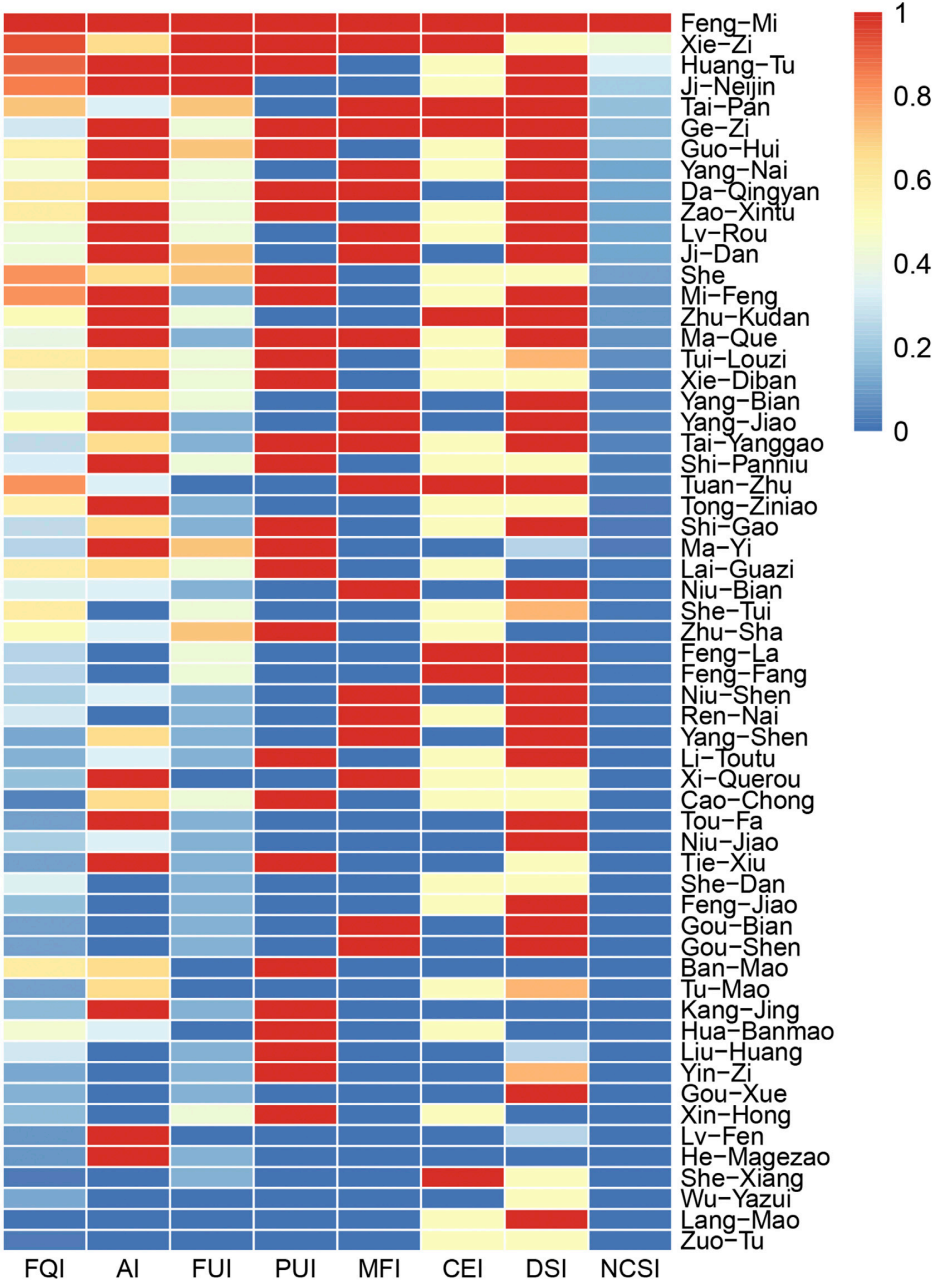
Quantitative evaluation of animal and mineral medicines in the study area. (FQI is the frequency of quotation index; AI is the availability index; FUI is the frequency of utilization index; PUI is the parts used index; MFI is the multifunctional use index; CEI is the curative effect index; DSI is the drug safety index; NCSI is the cultural food significance index).

The fourth and least crucial tier (NCSI <1) comprises only 6 medicines. These medicines are infrequently utilized and currently not readily available, such as Moschus and Lang-Mao. This may also be correlated with the reliability of the information (Figure 7).

Among the 59 identified animal and mineral medicines, we did not encounter any relevant literature reports for 5 of them, including Zuo-Tu, Wu-Yazui, Lang-Mao, Li-Toutu, and Kang-Jing. This study may serve as inaugural documentation. While there are reports on some medicines, such as Tou-Fa, Tu-Mao, Tie-Xiu, and He-Magezao, they are seldom observed in clinical practice, and their modes of application differ. The local residents’ utilization techniques and the ailments they address vary significantly from those of other ethnic groups. Moreover, among the 59 medicines, few share the same efficacies and indications as traditional Chinese medicine or other ethnic medicines. The local residents’ application of these medicines is distinctly distinctive. For instance, they employ Feng-Mi to alleviate conditions such as oral ulcers and sores, use heated Lü-Fen for dog bites, and apply *sulfur* through burning for fumigation to address skin disorders, among other practices.

### Evaluation of the SWOT of animal and mineral-based medicines used by local residents

**FIGURE 8:**
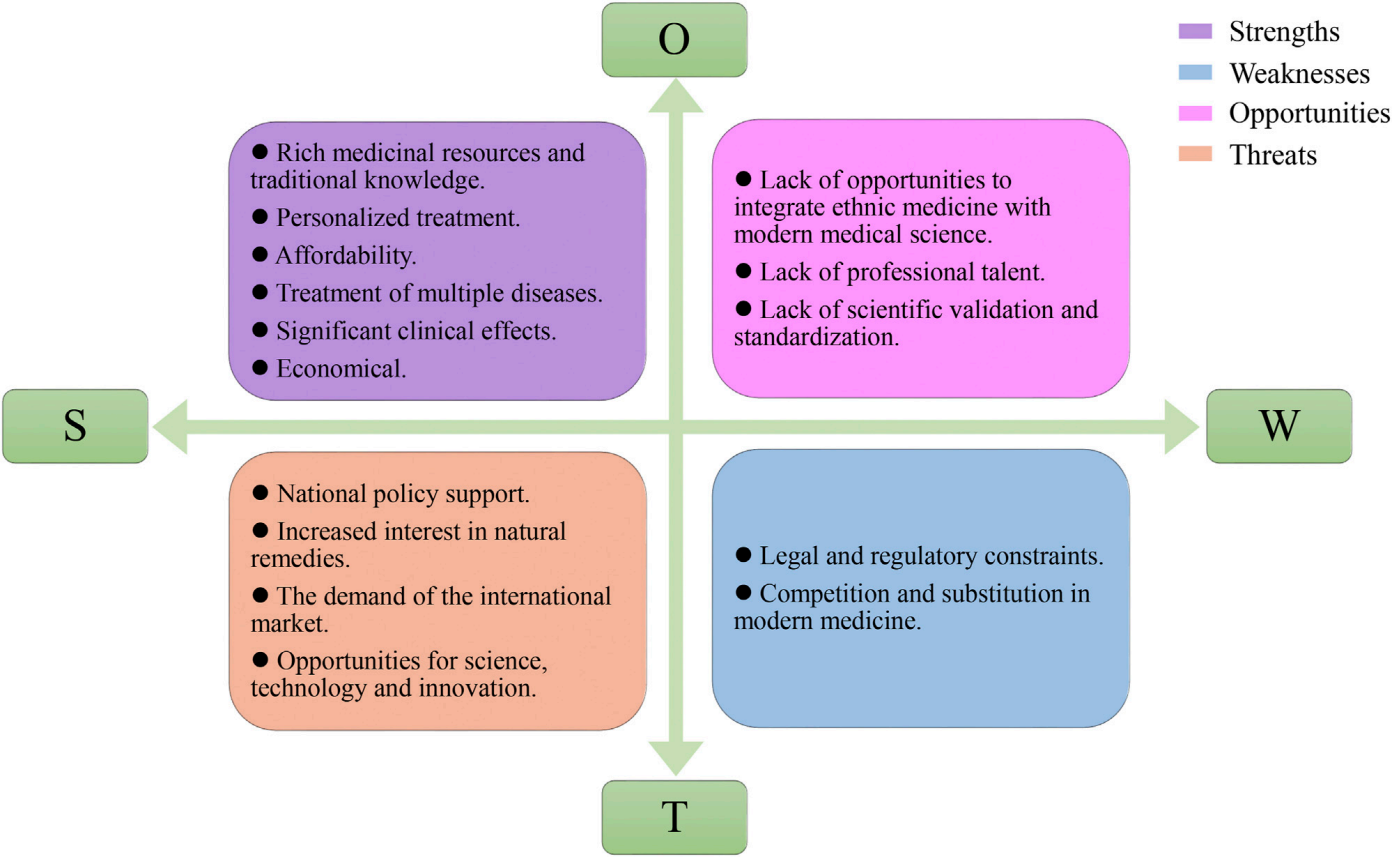
SWOT matrix for traditional medicines in the Gansu-Ningxia-Inner Mongolia intersection zone.

We conducted a comprehensive SWOT analysis to assess the potential of traditional medicines and traditional medical science within modern healthcare, presenting the findings in a SWOT matrix (Figure 8). The strengths of ethnic medicine are noteworthy. It employs natural medicinal herbs as primary treatment modalities, capitalizing on abundant and cost-effective resources. Accessibility and user friendliness are further advantages, coupled with minimal associated side effects. Ethnic medicine embraces a holistic approach, systematically dissecting the underlying causes of ailments before tailoring targeted treatments. Additionally, ethnic medicine exhibits diverse pharmacological effects, rendering it efficacious in treating a wide spectrum of diseases. For example, Chinese medicine not only alleviates pain and inflammation (Wang et al., 2018) but also addresses common afflictions such as colds and indigestion (Song et al., 2023; Wu et al., 2023). Tibetan medicine enhances immunity (Pu et al., 2021) and effectively addresses specific conditions, such as altitude sickness (Liu et al., 2018).

However, ethnic medicine confronts several weaknesses and potential threats that impede its advancement and widespread adoption. Foremost among these challenges is the paucity of scientific validation regarding its pharmacological effects and safety profile, prompting certain nations to impose restrictive measures and regulations. Another critical challenge lies in the erosion or incompleteness of certain traditional knowledge associated with ethnic medicine, a consequence of the relentless progress in modern medical science and technology. This has rendered the preservation and transmission of this knowledge an arduous task. Moreover, some patients may favor modern medical technology over ethnic medicine due to its perceived convenience and higher perceived efficacy.

Despite these challenges, ethnic medicine is poised to capitalize on several opportunities for growth and expansion in the current landscape. Notably, there is a discernible surge in the popularity and influence of ethnic medicine, both domestically and on the global stage. More individuals are seeking natural and alternative ways to enhance their overall health and well-being, which also means that we need more professionals to inherit and pass on ethnic medicine, as well as more efforts to scientifically validate its effectiveness. Another significant opportunity lies in the integration of ethnic medicine with modern medical science, augmenting its applicability and overall efficacy. It is therefore our recommendation to seize these opportunities to advance the global promotion of ethnic medicine. Concurrently, addressing its inherent weaknesses and potential threats necessitates concerted efforts in scientific research, policy advocacy, cultural preservation, and widespread public education.

### Modern pharmaceutical research

A comprehensive review of the chemical basis, biological activity, and applications of the 59 drugs employed in traditional Chinese medicine and other ethnic minority groups was conducted (Table 3). These medicines predominantly exhibit anti-inflammatory (Hossain et al., 2022), antioxidant (Tang et al., 2023), antibacterial (Zhao and Li, 2017), antitumor (Xia and Zhou, 2019), and analgesic effects (Gu et al., 2021), among others. Within the cohort of 59 traditional animal and mineral-based medicines utilized by local residents, no pertinent literature documentation was found for 5 medicines, including “Zuo-Tu”. Additionally, distinctions in application methods were identified between specific drugs and traditional ethnic medicines (including traditional Chinese medicine), such as *Moschus*. Although *Moschus* is a precious medicine employed in traditional Chinese medicine for both internal and external use, local residents predominantly use it for treating various unidentified swellings and lumps. Rather than ingesting *Moschus* directly, they store it in a glass jar containing several sewing needles, known as “yangzhen”. To address blood stasis, pain, and swelling, needles are employed to create circles around the affected area followed by multiple cross-cuts to stimulate surface bleeding. This approach mirrors the efficacy of traditional Chinese medicine in promoting blood circulation, dispelling blood stasis, and alleviating pain, as outlined in the Chinese Pharmacopoeia (Chinese Pharmacopoeia Commission, 2020).

**TABLE 3:** General information on animal and mineral-based medicines in the study area.

Local name	English name	Representative chemical components	Bioactivity	Representative drugs	References^1^
Zhu-Kudan	Pig’s bitter gall	Hyocholic acid, Hyodeoxycholic acid, Bile acid, Bilirubin	Antibacterial, Anti-inflammatory, Analgesic	Suis fellis Pulvis, Pig bile Paste	Jiang and Zhu (2019)
Feng-Mi	Honey	Caffeic acid, Quercetin, Bee antimicrobial peptides	Antioxidant, Anti-inflammatory, Antibacterial	Anti-cough and Asthma Oral Liquid, Feng-Mi Mouthwash	Hossain et al. (2022)
Feng-La	Beeswax	Higher fatty acid, Carbohydrate, Esters, Dotriacontanol, Triacontanol	Hypolipidemic, Antioxidant, Anti-thrombotic	Propolis chromium soft Capsules	Theodoraki et al. (2017)
Feng-Fang	Nidus vespae	Amino acid, Peptides, Organic acid, Phenols, Volatile oil	Anti-inflammatory, Analgesic, Antibacterial, Anticancer activity	Nidus Vespae Suppository	Guo et al. (2023)
Feng-Jiao	Propolis	Flavonoids, Phenolic acid, Terpene, Carbohydrate, Amino acid	Anti-inflammatory, Antibacterial, Antiviral	Propolis chromium soft Capsules	Peixoto et al. (2022)
Mi-Feng	Bee	Phospholipase A2, Hyaluronidase, Melittin, Apamin	Anti-inflammatory, Antibacterial, Anti-aging	Bee Venom Plastics	El-Seedi et al. (2020)
Hua-Banmao	Mylabris	Cantharidin, Cyclo-(L-proline-L-alanine), Cyclo-(R-proline-R-leucine)	Antitumor	Compound Banmao Capsules	Xia and Zhou (2019)
Xi-Querou	Magpie’s Meat	Protein, Carbohydrates, Inorganic salts, Vitamins, Fat	Improving the body’s immunity, Diuresis, Lowering body temperature	Muxiang Shunqi Pills, Niuhuang Shangqing Pills	Peng and Xia (2018)
Wu-Yazui	Crow’s beak	——	——	Wujin San	——
Tu-Mao	Rabbit hair	Cellulose, Lignin	Anti-pathogenic microorganism, Anti-inflammatory, Hemostatic	——	——
Xie-Diban	Pillbug	Sterols, Alkaloids compounds, Organic acids, Lipid compounds	Antitumor, Anticoagulation, Analgesic, Anti-inflammatory	Shufu Tablets	Duan et al. (2017)
She-Dan	Snake gall	Bile acid, Bilirubin, Cholesterol	Antitussive, Expectorant, Antitussive effects	Shedan Chenpi Tablets, Niuhuang Shedan Chuanbei Tablets	Chen et al. (2019)
She-Tui	Snake skin cast off during molting	Ossein, Amino acid, Fatty acids, Sterols	Antibacterial, Anti-inflammatory	Snake-shed ash combined with methotrexate Tablets	Zhao and Li (2017)
She	Snake	Cholesterol, Taurine, Oleic acid, Linoleic acid, Arachidonic acid	Anti-inflammatory, Anti-thrombotic, Anti-Cancer	Anticoagulant agents, Zhenzhu Qishe Oral Liquid	Zhang et al. (2021)
Tai-Yanggao	Fetal lamb	Protein, Amino acid, Trace elements, Phospholipids, Lipopolysaccharide, Vitamins	Enhancement of immune function, Anti-aging	Quanyang Pills	Yang (2018)
Yang-Nai	Goat’s colostrum	Lactose, Triglycerides, Whey protein	Antioxidant, Immune regulation, Intestinal flora regulation	Goat milk Powder	Zhao et al. (2019)
Yang-Bian	Penis and testis of a goat	Progesterone, Estradiol, Testosterone Propionate	Enhancing sexual function, Strengthens the muscles and bones	Butianling Tablets, Sanbian Capsules	Hu (2000)
Yang-Shen	Goat’s renal	Protein, Carbohydrates	Enhancing kidney function, Supplementation, Diuresis	White Lamb Kidney Soup, Acorus calamus Pills	Chen et al. (2023)
Yang-Jiao	Goat’s horn	Protein, Peptides, Complex Amino acid, Lipids	Sedation, Hypnosis, Anti-convulsive, Antipyretic	Compound claw Tablets, Compound claw Granules	Zhu et al. (2023)
Tai-Pan	Placenta	Hyaluronidase, Fibrinogen stability factor	Hemostatic, Antiplatelet aggregation, Anti-aging	Placental tissue Injection, Placenta Tablets	Tong et al. (2022)
Zuo-Tu	Loess	——	——	——	——
Ren-Nai	Breast milk	Lecithin, Oxalic acid, Cystine, Tryptophan	Promote hematopoietic function, Relieves dry skin mucous membranes	——	Cimmino et al. (2023)
Tou-Fa	Hair	Calcium ion, Iron ions	Hemostatic, Coagulation	Cuisheng Powder, Huiyan Paste	Liu et al. (2020)
Tong-Ziniao	Children’s urine	Urea, Sodium chloride, Potassium, Uric acid, Creatinine	Heat clearing, Sedation	White Plaster, E-ai Pills	Xu (2015)
Ban-Mao	Cantharides	Canthaxanthin, Cantharidin, Chitin	Antibody activity, Anti-cervical cancer	Boyun Ointment	Wang (2019)
Shi-Panniu	Dung beetle	Molossusamide A (1), Molossusamide B, Molossusamide C, Dung beetle toxin	Anti-inflammatory, Antitumor, Anticoagulation	Huoxue Xiaoying Tablets	Zhang et al. (2019)
Cao-Chong	Paramecium caudatum	Palmitic acid, Decanoic acid, Cholesterol, Antibacterial peptide	Antitumor, Antibacterial	Grub Eye Drops	Zhang (2021)
Tuan-Zhu	Honey badger	Glyceryl Ester Compounds, Fatty acids	Anti-inflammatory, Antibacterial, Anti-pathogenic microorganism, Promotes skin regeneration	*Meles meles* oil Applicator	Cao (2013)
Ma-Que	Sparrows	Protein, Fat, Inorganic salts	Enhancing immunity function	Maque nourishing Pellets	Wang (2023)
Lü-Fen	Feces of donkey	Protein, Fatty acids, Organic acid, Cellulose, Hemicellulose, Lignin	Diuresis, Sedation, Anti-convulsive, Anti-anxiety, Anticoagulation, Improve blood circulation, Inhibition of gastrointestinal smooth muscle	Erhui Powder, Heilong Pills	——
Lü-Rou	Flesh of donkey	Amino acid, Hexanal, Unsaturated fatty acid	Antioxidant	Sanshen Pill, Colla corii Asini	Wang and He (2021)
She-Xiang	Moschus	Polypeptide from Moschus SXP4, Muscarinone, Cholest-5-en-3β-ol, 5α-androstane-3, 17-dione	Anti-inflammatory, Antibacterial	Shexiang Baoxin Pills	Lv et al. (2022)
Lang-Mao	Wolf hair	——	——	——	——
He-Magezao	Tadpoles	17a, 20a- dihydroxy -4-pregnen-3-one	Anti-Cancer	Guanyin Dew, Tadpole Detoxification Powder	Yang et al. (2003)
Tui-Louzi	Antlion	Long-chain fatty acids, Fatty acid esters, Peptides, Flavonoids, Alkaloids	Anti-thrombotic, Anticoagulation, Analgesic, Anti-inflammatory	Jinshaniu Huashi Tablets	Gu et al. (2021)
Ji-Neijin	Membranes of chicken gizzards	Gastrin, Galli Gigerii Endothelium Corneum polysaccharides, Tyrosine	Promoting the gastrointestinal peristalsis function, Antilithic	Child compound Endothelium Corneum, Galli Gigerii Endothelium Corneum decoction Pieces	Wu et al. (2020)
Ji-Dan	Eggs	Carotenoids, Active peptide	Antioxidant, Bactericidal, Hypoglycemic	Eucommia herbal Eggs, Herbal Eggs	Xue (2010)
Niu-Bian	Bull’s penis	Cholesterol, Testosterol, Dihydrotestosterone, Estradiol	Enhancing sexual function, Relieve fatigue	Bullwhip Cream	Xu et al. (2019)
Niu-Shen	Ox kidney	Aldosterone, Adrenalone, Desoxycorticosterone	Enhancing sexual function, Relieve fatigue	Shenrongsanshen Capsule, Jiuwei Shenrong Capsules	Hu and Hao (1989)
Niu-Jiao	Ox horn	Horn Fiber, Sterols, Guanidine	Analgesic, Anti-inflammatory, Anti-infection	Niujiao Dihuang Decoction	Tang et al. (2023)
Gou-Bian	Dog’s penis	Androgen, Protein, Fat	Enhancing sexual function	Sanbian Wenyang Capsules	Zhao et al. (2016)
Gou-Shen	Dog kidney	Androgen, Protein, Fat	Enhancing sexual function	Haima qiangshen Pills	Chen et al. (2023)
Gou-Xue	Dog’s blood	Protein, Water	Immune Enhancement, Blood enrichment, Sedation, Anti-arrhythmia, Antibacterial, Anti-inflammatory	Yangqi Shengling Dan	——
Ge-Zi	Pigeons	Protein, Lecithin, Calcium	Enhancing immunity function	Compound Pigeon Egg Yigan Huayu Pills	Science (2019)
Ma-Yi	Ant	Acetyldopamine, Trolline, Oleic acid	Anti-inflammatory, Analgesic, Anti-aging, Regulate blood sugar	Compound black ant capsule, Tianyi Astragalus Granules	Ma (2015)
Xie-Zi	Scorpions	Scorpio toxins, Polypeptide, Taurine	Antibacterial	Zaizao Pills, Da-Huoluodan	Xu (2002)
Lai-Guazi	Toad	Bufadienolides, Indole alkaloid, Bufogargarizanines, Steroids	Antitumor, Anti-inflammatory, Immune regulation	Toad Skin Tablets, Toad Cream	Liu et al. (2023a)
Zhu-Sha	Cinnabaris	Mercury Sulfide, Mercury carbonate, Mercury acetate	Sedation, Hypnosis, Antiviral, Antibacterial	Zhusha Anshen Pill, Angong Niuhuang Pills, Bawei Qinpi Pills	Liu et al. (2021)
Xin-Hong	Arsenolite	Arsenic trioxide	Antitumor	Ferula Pills	Guo et al. (2021)
Shi-Gao	Gypsum Fibrosum	Calcium sulfate dihydrate (CaSO_4_·2H_2_O), Ulfide and other trace elements	Heat clearing, Sedation, Anti-inflammatory, Promotes wound healing, Anti-inflammatory, Hemostatic	Xiaoqinglong plus gypsum Decoction, Baihu Decoction	Shi et al. (2021)
Li-Toutu	Plow soil	——	——	——	——
Tie-Xiu	Rust	Iron oxide (Fe_2_O_3_)	Anti-inflammatory, Antibacterial, Antiviral, Sedation	Tiexiu Liuhuang Powder	Tan (2003)
Zao-Xintu	Oven earth	Silicic acid (H_2_SiO_3_), Aluminum oxide (Al_2_O_3_), Iron oxide (Fe_2_O_3_)	Sedation, Anti-emesis, Anti-caries	Fulonggan Pills, Huangtu Decoction	Wang et al. (2021)
Guo-Hui	Pot bottom ash	Calcium oxide (CaO), Magnesium oxide (MgO), Silicic acid (H_2_SiO_3_), Iron oxide (Fe_2_O_3_)	Antibacterial, Anti-inflammatory	Baicaoshuang hemorrhoid Ointment	Zhang et al. (2022a)
Kang-Jing	Tar	——	——	——	——
Huang-Tu	Loess	Silicic acid (H_2_SiO_3_), Aluminum oxide (Al_2_O_3_), Iron oxide (Fe_2_O_3_)	Analgesic	Huangtu San, Huangtu Pills	——
Da-Qingyan	Carnallite	Sodium chloride (NaCl)	Antibacterial, Anti-inflammatory	Dahuang Huayu Pills, Qinglin Pills	Zhou and Xu (2016)
Liu-Huang	Sulphur	Sulfur S)	Antibacterial, Stimulation of the intestinal wall, Relieving diarrhea	Sulfur Ointment	Tan (2003)
Yin-Zi	Silver	Silver (Ag)	Antibacterial	Yinxie Capsules, Anshen Dingzhi Pills	Gao et al. (2005)

Notes: ^1^Wu-Yazui, Tu-Mao, Zuo-Tu, etc. are mostly used as medicines in local remedies, which have been preserved through oral transmission from generation to generation. And some of them are also widely used in traditional practices and are believed to have certain pharmacological effects. However, the efficacy of these medicines is only based on traditional knowledge and experience, and these views lack the support of scientific research, which is needed to verify their pharmacological activities. Therefore, they are denoted by "-" in Table 3.

## Discussion

Animal and mineral-based medicines, while less commonly utilized than plant-based remedies, possess unique therapeutic properties. In the arid region where the Loess Plateau intersects with the desert in western China, local communities have demonstrated ingenuity in harnessing the medicinal potential of animal waste, emphasizing resourcefulness in an environment with limited resources.

### The role of animal and mineral-based medicines in traditional medicine

Traditionally, animal-based medicines constitute approximately one-ninth of the total medicinal inventory, with mineral-based remedies even rarer, accounting for merely one percent of the pharmacopeia (Dong et al., 2023; Lu et al., 2023; Meng et al., 2023; Xu et al., 2023; Zheng et al., 2023). Nonetheless, our research conducted in the ethnically diverse crossroads of Gansu, Ningxia, and Inner Mongolia reveals a distinctive usage pattern. Local inhabitants employ both animal and plant medicines, with mineral medicines comprising approximately one-fourth of the combined total (Xie et al., 2023). This marked departure from traditional ratios underscores the widespread discovery and application of medicinal properties derived from animal resources by local communities. This shift can be attributed, in part, to the region’s resource scarcity, as arid environments exhibit significantly lower species diversity and resource reserves in comparison to southern China, thereby constraining access to plant-based resources (Bai et al., 2011; Li, 2013).

### Characteristics of medicinal animal and mineral resources

Our investigation uncovered a fascinating aspect of traditional medicine practiced by diverse ethnic groups across various regions (Bai, 1999). Beyond the distinct regional attributes of plant-based remedies, animal-based medicines also exhibit noteworthy regional characteristics. The animal-based medicinal resources employed by local inhabitants are primarily suited for arid climates rather than humid climates. For instance, in the Chinese martial arts literature, the “Five Poison Sect” employs various venomous insects, such as *Scorpios* and *Scolopendra*, cohabiting in the same container to evolve and yield the highly toxic “Gu” insect, aiding them in battle (Jin, 2010). Nevertheless, in reality, we observed that Scorpios are prevalent in the dry northern regions of China, while *Scolopendra* thrive in the humid south, making interactions unlikely due to their disparate habitats. Another Chinese folklore, “The Donkey from Guizhou,” (Liu, 2021) similarly underscores the regional characteristics of animal-based medicinal resources. This narrative suggests that thousands of years ago, there were no donkeys in the southwestern province of Guizhou, and even today, none were found during our investigation. However, the tiger, another key figure in the tale, once held traditional and esteemed medicinal value for ethnic minorities in Guizhou.

### Characteristics of traditional medicine culture of local residents

The utilization of animal and mineral-based medicines by local residents is widespread, and local remedies exhibit both diversity and distinctiveness. The amalgamation of various ethnic cultures has given rise to a distinctive medical culture in the region. However, the transmission of traditional medical knowledge primarily occurs orally and through apprenticeship, emphasizing a blend of practical application and experiential learning.

Broadly, the traditional medicine culture in the region is rooted in traditional Chinese medicine, enriched by an overlay of mystical shamanic practices. Often, treatments integrate both medicines and talismans, particularly in cases of mental health disorders. For instance, employing *Cinnabaris* in talismanic drawings has shown a discernible therapeutic effect in the treatment of mental illnesses. Additionally, there are simpler treatments, such as acupuncture, gua sha, and massage (Samuels et al., 2008; Feng and Dong, 2020).

## Conservation of animal resources through traditional practices by local residents

**FIGURE 9:**
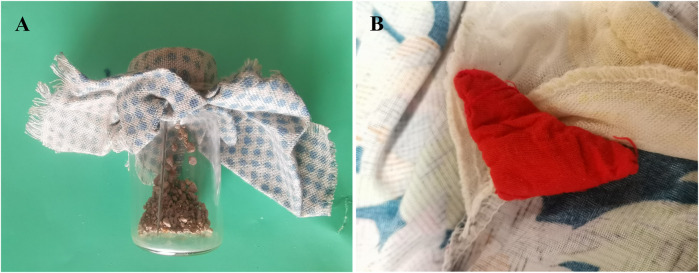
Talismans made from mineral medicine by local residents believed to have the effect of exorcism but were actually used for calming and tranquilizing. **(A)** *Moschus* stored in a glass bottle and placed beside the pillow when sleeping. **(B)** *Cinnabaris, Realgar*, and other minerals sewn into cotton cloth and worn on the body.

Of the 59 varieties of animal-based medicine employed by local residents, *Moschus* holds the highest regard. Residents can only provide small quantities of samples, carefully preserved for over half a century. Regrettably, local *Moschus moschiferus* Linnaeus (*M. moschiferus* L.), known as “Xiangzhang” in the area, has become extinct. Another prized animal medicine, Scorpio, has faced rampant exploitation due to its soaring market value. In response, local authorities have implemented stringent regulations to safeguard this species (China Food and Drug Administration, 2006), leading to instances of legal action against residents involved in unauthorized capture. It is worth noting that wildlife conservation extends beyond only rare species in the region. In contrast, awareness of mineral medicine conservation among local residents remains limited. On the one hand, the variety of mineral medicines is limited, and valuable specimens such as *Cinnabaris* and *Realgar* are predominantly acquired from Chinese medicine stores. The majority of other mineral medicines are not considered scarce resources (Figure 9).

### Current status of local residents’ application and inheritance of traditional knowledge of animal and mineral-based medicines

The application of traditional medicine among local residents carries a certain mystique. However, with the advancement of technology and traditional Chinese medicine, this mystique has gradually waned in market appeal (Kiernan, 1978; Xie and Li, 2022). Following the passing of the older generation of rural doctors with distinct shamanic characteristics, the utilization of traditional medicine has seen a sharp decline. This decline is particularly pronounced among young residents who lean toward trust in Chinese and modern medicine. They encounter difficulty in embracing the use of certain traditional remedies, such as Zuo-Tu and Tong-Ziniao. This dynamic, on the one hand, reflects the advancing civilization of local residents. On the other hand, it has also resulted in the erosion of significant longstanding cultural knowledge, including that of traditional medicine (Gao, 2007; Qin et al., 2011).

### Suggestions for protecting and promoting local traditional ethnic medicine knowledge

To safeguard and promote traditional ethnic medicine knowledge, we propose the following measures. First, it is imperative to enhance the conservation of wild resources used in animal-mineral medicine while also nurturing the cultivation and development of medicinally valuable plants. Second, there is a pressing need to amplify the promotion and dissemination of traditional ethnic medicine knowledge. This effort is instrumental in fostering a broader understanding and recognition of its cultural significance. Third, we advocate for the reinforcement and continuation of efforts to cultivate talent in the field of traditional ethnic medicine. This ensures the effective transmission and evolution of this vital knowledge. Fourth, harnessing the strengths of modern medicine is paramount. This can be achieved through the integration of traditional ethnic medicine with modern practices, elevating the application and efficacy of animal-mineral medicine. Last, establishing a comprehensive management system for the protection and utilization of medicinal resources is of paramount importance. Such a system will oversee the collection, processing, and utilization of animal and mineral-based medicine, thereby ensuring the sustainable use of these resources.

### Ethical and safety issues of animal-and mineral-based medicine in traditional Chinese medicine

In China, TCM has a long history and enjoys wide popularity and governmental support. China has established a comprehensive legal framework to foster the development and inheritance of TCM (Xue et al., 2023). Currently, TCM has been extensively applied and developed both within China and internationally. A large number of animal-based medicines are used in TCM, which inevitably raises animal ethics issues (Chen, 2020). Most of these medicines are derived from dead animals or from legally hunted animals rather than from live animals. These practices, although ethically controversial, do not involve the abuse or torture of live animals. To promote the sustainable practices of local precious animal medicinal resources and to raise the education and awareness of animal welfare, we communicated and exchanged views with the local government officials from the agricultural and forestry departments, as well as the township level, during our investigation process. We aimed to attract the attention of the local government to the animal medicinal resources through various channels, and to improve animal welfare and human health.

Another interesting and important topic is the toxicity issue of mineral medicines. TCM has developed a unique application of mineral medicines, especially using some toxic minerals, forming a distinctive knowledge system and safety procedures (Lu et al., 2017; Wang et al., 2023). For example, arsenic trioxide, a highly toxic mineral, is used for specific medical purposes. Extensive modern pharmacological research has confirmed its effectiveness and safety under certain conditions (Nithyananthan and Thirunavukkarasu, 2020; Guo et al., 2021). Of course, the use of these toxic mineral medicines also requires strict adherence to the correct dosage, compatibility, preparation, administration and storage methods to avoid adverse reactions or poisoning events.

### SWOT strategy formulation

Based on the results of the SWOT analysis, the corresponding strategies are proposed as follows: First, give full play to the strengths, strengthen the education and training of ethnic medicine, cultivate more experts and researchers of ethnic medicine, strengthen scientific research and clinical practice, and improve the scientific validity and credibility of ethnic medicine. Second, overcome the weaknesses, strengthen the scientific verification and standardization of ethnic medicine, establish a scientific evaluation and certification system, strengthen the protection and inheritance of traditional knowledge, improve the clinical application of ethnic medicine, strengthen the cultivation of talent, and improve the talent pool and quality of ethnic medicine. Third, seize opportunities to strengthen international cooperation and exchanges, promote the advantageous features of ethnic medicine, attract more international students and research institutes to participate, and carry out international exchanges and exhibitions to improve the international influence of ethnic medicine. Fourth, cope with threats and strengthen legal and regulatory protection of ethnic medicine to ensure its legal status and rights and interests, enhance public recognition and trust in ethnic medicine and strengthen publicity and education efforts.

## Conclusion

In our investigation, we documented and compared the traditional knowledge and use of 47 types of animal medicines and 12 types of mineral medicines among different ethnic groups in the Gansu-Ningxia-Inner Mongolia region of China. Our findings reveal the rich and diverse aspects of the local traditional medicine culture, as well as the current status and challenges of the local animal and mineral resources. One of the major challenges is the use of toxic minerals in traditional medicine, which poses serious health and environmental risks. Therefore, we suggest that a critical appraisal of the safety and efficacy of these substances is urgently needed to ensure the ethical and sustainable use of traditional medicine. Our study provides valuable baseline data for the conservation and development of these traditional medicine cultures, which are part of the global heritage of human civilization. However, we acknowledge that our study is limited by the lack of comprehensive evidence on the clinical value and biological activity of these medicines. Therefore, we propose that future research should focus on the material foundation, pharmacological effects, clinical trials, and safety assessment of these medicines. TCM has become a promising industry in China and the world, thanks to its unique advantages and policy support, but it also faces some internal weaknesses and external threats. In order to integrate TCM into the health service industry, we recommend that more efforts should be made to enhance international cooperation and exchange, learn from international experience and advanced technology, promote the internationalization of ethnic medicine, establish and implement standards to improve the quality and safety of ethnic medicine, introduce modern technology and management methods to promote the modernization of ethnic medicine, and encourage innovative research and practice to promote the innovation of ethnic medicine.

## Data Availability

The original contributions presented in the study are included in the article/Supplementary Material, further inquiries can be directed to the corresponding authors.

## Abbreviations

NCSI, cultural food significance index; FQI, Frequency of quotation index; AI, Availability index; FUI, Frequency of utilization index; PUI, Parts used index; MFI, Multifunctional use index; CEI, Curative effect index; DSI, Drug safety index.
